# Application of Wireless EMG Sensors for Assessing Agonist–Antagonist Muscle Activity During 50-m Sprinting in Athletes

**DOI:** 10.3390/s25206395

**Published:** 2025-10-16

**Authors:** Kanta Yokota, Hiroyuki Tamaki

**Affiliations:** 1 Graduate School of Physical Education, National Institute of Fitness and Sports in Kanoya, Kanoya 891-2311, Japan; m257006@sky.nifs-k.ac.jp; 2 Department of Sports and Life Science, National Institute of Fitness and Sports in Kanoya, Kanoya 891-2311, Japan

**Keywords:** electromyography, muscle activity, ground reaction force, sprint running, cross-correlations, lissajous figure

## Abstract

**Background:** Wireless surface electromyography (sEMG) enables the investigation of neuromuscular control in realistic sports settings; however, ensuring reliable signal acquisition during sprinting remains challenging. This study examined the feasibility of continuous wireless EMG recording in sprinting athletes and evaluated their agonist–antagonist coordination patterns. **Methods:** Ten trained sprinters performed four maximal 50-m sprints on a force plate–equipped track. sEMG was recorded from the biceps femoris (BF), rectus femoris (RF), soleus (Sol), and tibialis anterior (TA) under two receiver configurations: fixed-receiver condition (FRC) and mobile-receiver condition (MRC). Integrated EMG, kinematics, and cross-correlation analyses were performed on a stride-by-stride basis. **Results:** Continuous high-quality EMG was feasible under MRC, highlighting the practical importance of maintaining receiver proximity in sprint experiments. BF activity during the late swing phase correlated positively with sprint velocity, supporting the performance relevance of pawing. BF/RF interactions varied substantially across individuals, whereas Sol/TA were consistently coactivated, indicating ankle stabilization. **Conclusions:** Wireless EMG enables reliable in-field monitoring of sprinting athletes, revealing both individualized and shared coordination strategies relevant to performance and injury prevention in athletes.

## 1. Introduction

Recent advances in wireless sensor technology, including surface electromyography (sEMG), have significantly expanded the possibilities for recording physiological and biomechanical data in naturalistic and high-performance sports settings [1]. Traditionally, sEMG systems were wired, limiting their application to laboratory-based experiments because of restrictions on movement and measurement range. However, the development of miniaturized and wireless systems has overcome many of these limitations, making it possible to record muscle activity during full-body dynamic tasks, such as sprinting, in real athletic environments. These wireless technologies have led to increased research on sprinting biomechanics, not only in terms of kinematics and kinetics but also in terms of neuromuscular control [2]. Earlier studies often employed treadmills because of the physical constraints of wired systems [3], but recent studies have demonstrated the feasibility of field-based EMG recordings during sprinting on official 400-m tracks using wireless systems [4,5,6].

Among the various aspects of muscle function during sprinting, the interaction between agonist and antagonist muscles has drawn increasing attention. This interest stems from both performance and injury prevention perspectives [7,8,9]. From a performance standpoint, efficient coordination between these muscle groups optimizes force transmission and minimizes unnecessary co-contraction, thereby enhancing the sprinting speed and movement economy [10]. From an injury prevention perspective, appropriate levels of co-contraction can stabilize joints under high loads, but excessive antagonist activation may elevate the strain on muscle–tendon units. This is particularly relevant for the hamstrings during the late swing phase when they undergo rapid eccentric loading and when most sprint-related hamstring injuries occur.

During typical locomotion, the alternating activation of agonist and antagonist muscles supports efficient and smooth movement. This coordination is mediated by reciprocal inhibition, a neural mechanism in which the activation of one muscle suppresses the activity of its antagonist. However, spinal reciprocal inhibition decreases significantly as the movement speed increases [11]. Such a reduction in inhibition may contribute to a shift toward co-contraction under high-speed or high-load conditions, where agonist and antagonist muscles are simultaneously activated [12,13,14,15]. While co-contraction enhances joint stability and may help prevent injury, it can also diminish agonist force output, thereby reducing movement efficiency [16]. This trade-off is particularly critical in sprinting, where extremely high velocities and impact forces occur within very short timeframes. Despite its relevance to performance optimization and injury prevention, the neuromuscular coordination between agonist and antagonist muscles during maximal sprinting, especially over longer distances such as 50 m, is still not fully understood. This knowledge gap arises not only from the technical challenges of collecting precise, continuous physiological and biomechanical data under genuine sprint conditions but also from the complex and highly individual-specific nature of neural control strategies during maximal effort.

The present study aimed to characterize lower limb neuromuscular coordination during maximal sprinting in a field-based environment, with a particular focus on the interaction between agonist and antagonist muscles. A wireless EMG sensor was used to capture muscle activation patterns under realistic sprint conditions. In addition, we sought to examine the individual-specific characteristics of these coordination patterns, acknowledging the highly individual nature of neural control strategies at maximal effort. Furthermore, we addressed a methodological issue concerning the feasibility of continuously recording EMG signals over the full sprint distance in a field-based environment.

## 2. Materials and Methods

### 2.1. Participants

Ten sprinters participated in this study (nine males: 21.8 ± 3.1 years, 1.72 ± 0.06 m, 67.7 ± 3.0 kg, one female: 22 years, 1.63 m, 59.3 kg). The participants specialized in sprint running event (100-m, 200-m, 110-m Hurdle, 400-m Hurdle). This study was approved by the research ethics committee of the National Institute of Fitness and Sports in Kanoya (23-1-6). All participants were free of any history of lower extremity injury or neurological disorders at the time of the test.

### 2.2. Experimental Protocol

The sprint trials were conducted indoors at the SPORTEC Sports Performance Research Center (NIFS in Kanoya, Japan) on a 50-m straight tartan track equipped with an array of 54 force plates embedded along the entire length of the track. After completing a standardized warm-up, the participants performed four 50-m maximal sprints from a crouched position using starting blocks. Each sprint trial was separated by a rest interval of at least five minutes to minimize fatigue and ensure consistent maximal performance. During each sprint, surface electromyographic (sEMG) signals from the lower limb muscles and ground reaction forces were recorded simultaneously. All signals were time-synchronized to ensure the precise alignment of neuromuscular and biomechanical data for subsequent analysis. To evaluate the optimal signal acquisition conditions, two sprint trials were conducted under a fixed-receiver condition (FRC), where the wireless receiver unit (Wave Plus, Cometa, Italy) was placed at the 25-m midpoint of the 50-m track. In the other two trials, measurements were performed under a mobile-receiver condition (MRC), with the receiver unit dynamically following the runner at an approximate distance of 5–7 m to ensure continuous data transmission throughout the entire sprint distance.

### 2.3. Instrumentation and Data Collection

Surface electromyographic (sEMG) signals were recorded from the tibialis anterior (TA) and soleus (Sol), rectus femoris (RF) and biceps femoris (BF) muscles of the right leg using wireless Cometa Pico EMG sensors (Cometa Systems, Milan, Italy). These muscles were selected because they represent functionally antagonistic pairs at the thigh (BF–RF) and shank (Sol–TA), allowing the analysis of reciprocal muscle activation patterns between agonist and antagonist groups during sprint running. Prior to electrode placement, the skin was shaved if necessary, lightly abraded, and cleansed with alcohol to ensure low impedance (<5 kΩ). Each sensor unit incorporated its own integrated transceiver and transmitted data via Bluetooth transmission to a common receiver. The hardware weight of each sensor unit was approximately 7 g, and the device provided continuous operation for approximately 12 h on a full charge. No participant reported disturbance or performance impairment caused by the sensor units.

Conductive-gel-based disposable Ag/AgCl surface electrodes with a 8-mm diameter (Cardinal Health, Dublin, OH, USA) were applied in a bipolar configuration over the muscle bellies, aligned parallel to the muscle fiber direction, in accordance with the SENIAM guidelines [17]. The interelectrode distance was maintained at approximately 20 mm. The EMG signals were amplified and band-pass filtered (10–500 Hz). EMG signals were sampled at 2000 Hz to prevent aliasing and to capture high-frequency components during rapid, ballistic contractions. Although the main frequency content of surface EMG lies below ~500 Hz, oversampling at 2000 Hz provides adequate resolution for detecting rapid fluctuations and minimizes reconstruction errors. According to the Nyquist theorem, the sampling frequency should exceed at least twice the maximum frequency component of interest; in practice, 2–3 times higher frequencies are recommended to ensure adequate temporal resolution and to reduce aliasing risk [18]. This rationale is also in line with previous sprint EMG studies that adopted the same sampling rate [4,5,6].

GRF data were collected from a 50-m long force plate system (TF-90100, TF-3055, TF-32120, Tec Gihan, Uji, Japan) at a sampling rate of 2000 Hz. Data were collected using the “All-Plate Mode”, in which 50 force plates installed along the 50-m runway are CPU-integrated to function as one continuous force plate (1 m × 50 m). The remaining four plates at the starting line were used independently for the starting blocks (left and right foot) and hand placements, and were not included in the CPU integration.

**Figure 1:**
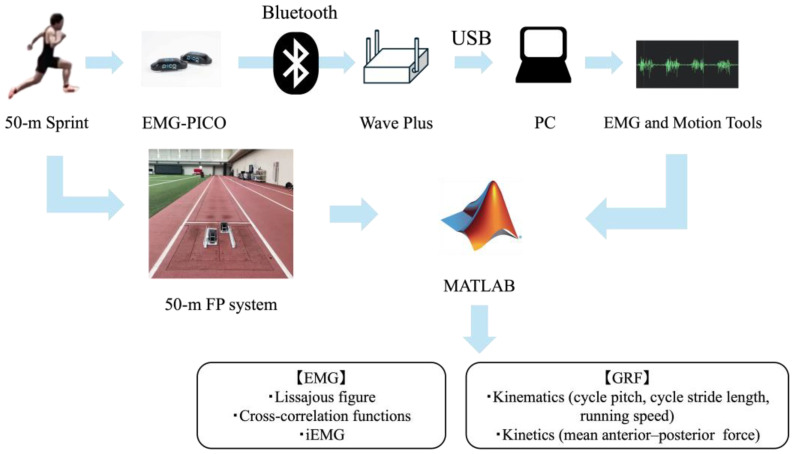
A series of methods for recording sEMG and GRF during 50-m sprint.

sEMG and GRF signals were synchronized using a transistor–transistor logic (TTL) trigger signal transmitted via a Bayonet Neill–Concelman (BNC) cable. The sEMG signal was collected using a wireless receiver unit (Wave Plus, Cometa, Milan, Italy) and transmitted to a PC via USB using proprietary acquisition software (EMG and Motion Tools 8.6.2.0, Cometa, Milan, Italy). To accommodate receiver mobility during overground sprinting, the Wave Plus receiver was carried by the examiner, following the participant. Because the receiver was bus-powered via USB, a 30-m active USB extension cable with a built-in signal repeater (KB-USB-R230; Sanwa Supply, Okayama, Japan) was used to prevent signal degradation. The Wave Plus also supports external synchronization via a 2.5 mm audio jack. For precise temporal alignment, a TTL pulse was generated using a starting pistol and transmitted simultaneously to both the Wave Plus and GRF systems via a 2.5 mm jack–to–BNC cable (Figure 1).

### 2.4. Data Analysis

The sEMG and GRF data were analyzed using MATLAB (The Math Works, Natick, MA, USA).

The sEMG signals were processed as follows: (1) bandpass filtering with a bandwidth of 20–500 Hz, (2) rectification, (3) smoothing with a time constant of 0.03, and (4) normalization based on the maximum amplitude during maximal voluntary contraction (MVC) (%EMG@MVC, i.e., EMG amplitudes expressed as a percentage of MVC). Following normalization, the EMG signals were time-integrated within each stride cycle to obtain the integrated EMG (iEMG), expressed in %MVC·s.

Agonist–antagonist muscle activity patterns were visualized using x–y plots based on the smoothed sEMG signals. The x- and y-axes of the plot represent the posterior (BF, Sol) and anterior (RF, TA) muscles, respectively, based on their anatomical locations. To assess the temporal relationship between agonist and antagonist muscle activity patterns, cross-correlation analysis was performed between the normalized iEMG signals of biarticular thigh and lower leg muscle pairs using the normalized cross-correlation function (xcorr, coeff) in MATLAB.

For each stride cycle, the BF and Sol signals were designated as the reference signals (first muscle), and the RF and TA signals were designated as the second muscle in each pair. The maximum cross-correlation coefficient (r) and its corresponding lag time (in seconds) were extracted, in addition to the coefficient at a lag of zero. A positive lag indicated that the activity of the second muscle (RF or TA) followed that of the first muscle (BF or Sol), whereas a negative lag indicated that the second muscle preceded the first muscle. The coefficient at lag zero was used as an indicator of the coactivation.

**Figure 2:**
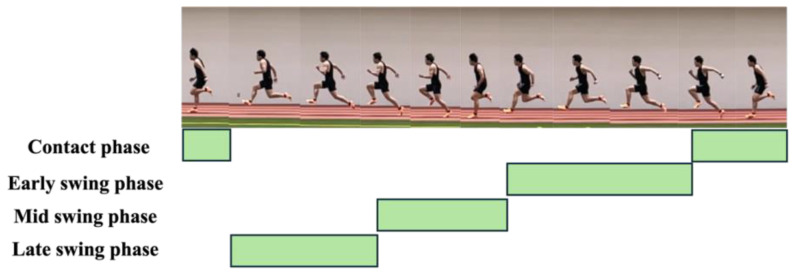
Each right-leg stride cycle was divided into four phases. The contact phase was defined as the duration from the foot strike to the foot-off. The swing phase was further divided into three subphases: early swing phase (from foot-off to contralateral foot strike), mid-swing phase (from contralateral foot strike to contralateral foot-off), and late swing phase (from contralateral foot-off to ipsilateral foot strike).

**Figure 3:**
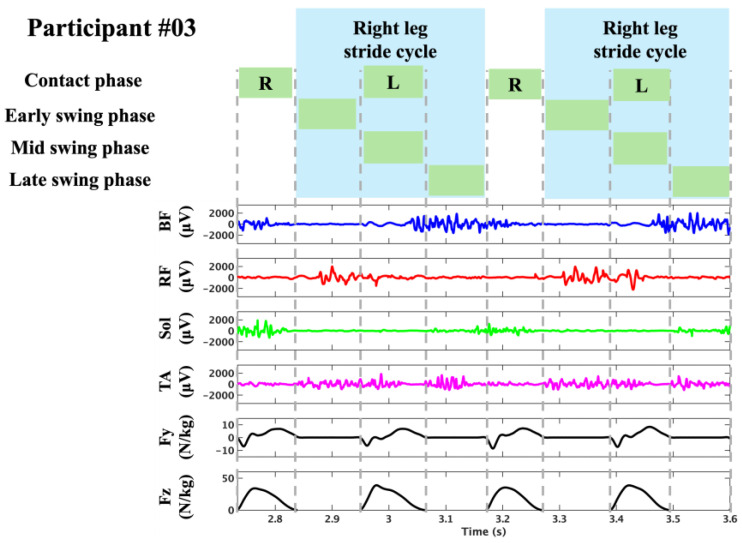
Representative recordings of sEMG (BF, RF, Sol, and TA) and ground reaction force (Fy: anterior–posterior; Fz: vertical) for participant #03. The gray dashed lines indicate the boundaries between the movement phases, and the blue-shaded region marks the stride cycle analyzed in this study.

GRF data were processed using a low-pass filter (The Math Works, Natick, MA, USA) with a cutoff frequency of 50 Hz, and a threshold of 20 N was applied to the vertical GRF component (Fz) to distinguish the foot strike, following previous studies [19,20]. In the present study, the foot strike was defined as the point at which Fz exceeded this threshold for 20 consecutive samples, and the foot-off was identified as the point at which this continuity ceased. The period between the foot strike and subsequent foot-off was defined as the ground contact phase, and its duration was defined as the contact time (CT). One lower limb stride cycle was defined as the interval from the foot strike of one leg to the subsequent foot strike of the same leg. The duration of this cycle was termed cycle time (1CTime). Each cycle was divided into a ground contact phase and a swing phase (Swing), with the swing phase further subdivided into early swing (ESwing), mid-swing (MSwing), and late swing (LSwing), resulting in four distinct phases. ESwing was defined as the period from foot-off of the ipsilateral leg to foot strike of the contralateral leg, MSwing, from contralateral foot strike to its foot-off, and LSwing, from contralateral foot-off to ipsilateral foot strike (Figure 2). Figure 3 present representative sEMG signals and GRF data aligned with specific stride cycle phases. This figure depicts the period from one right foot contact to the next occurrence of right foot contact two cycles later, thus covering two complete stride cycles. The duration of each was defined as the phase time. The horizontal component of the center of pressure (COP) was used to calculate the stride length (SL) and cycle time. For each cycle, the midpoint of the COP during the ground contact phase of the right leg was identified, and the average of 10 samples (five before and five after the midpoint) was computed [20]. SL was defined as the distance between the average COP position at the right foot strike and the average COP position at the subsequent right foot strike. The cycle time was defined as the elapsed time between two successive right-foot strikes. The cycle frequency (CF) was calculated as the reciprocal of 1Ctime, and the running speed (RS) was obtained as the product of SL and CF. The mean anteroposterior force (mAP), which has been reported in previous studies to be associated with sprint acceleration performance, was calculated [19]. For each step, the anteroposterior GRF component was averaged over the ground-contact phase and normalized to body mass (N/kg).

### 2.5. Statistical Analysis

Statistical analyses were performed using MATLAB (MATLAB ver. R2023a; The MathWorks, USA). Data are expressed as mean ± standard deviation (SD). To assess the within-rater reliability of the EMG measurements, intraclass correlation coefficients (ICC) were calculated based on a two-way mixed-effects model [ICC (3,1)], reflecting the consistency of single measurements across three repeated trials for each participant. ICC (3,1) was selected because it is appropriate when the same rater evaluates all measurements under fixed conditions, and when absolute agreement for single measurements is of interest. Following Carius et al. (2015) [21], reliability was interpreted as good (ICC ≥ 0.80), fair (ICC 0.60–0.79), or poor (ICC < 0.60). The ICC (3,1) for TA EMG amplitude at MVC was 0.964, with a 95% confidence interval ranging from −0.845 to 1.00, indicating good within-rater reliability.

Pearson’s correlation coefficients were calculated to examine the relationships between the integrated EMG (iEMG) activity of each muscle (BF, RF, Sol, and TA) and sprint performance variables, running speed (RS) and mean anteroposterior force (mAP).

The relationships between running speed and relative iEMG (%MVC·s) of each muscle (BF, RF, Sol, and TA) were examined using regression analyses. Both linear (Pearson’s correlation) and nonlinear (exponential and quadratic) models were tested. For linear models, Pearson’s correlation coefficients (r) and their significance levels were reported, whereas for nonlinear models the goodness of fit was evaluated by the coefficient of determination (R^2^). Model selection was based on both the magnitude of R^2^ and the inspection of residual plots. The model that achieved the highest R^2^ without systematic patterns in the residuals was considered the best fit for each muscle group.

Cross-correlation analyses were performed for each muscle pair (RF–BF and TA–Sol). EMG signals were segmented stride-by-stride throughout the 50-m sprint, and cross-correlations were computed for each stride. Both the peak correlation coefficient and the corresponding time lag were extracted as primary outcome measures, characterizing the strength and temporal characteristics of the agonist–antagonist muscle activation patterns. Statistical significance was set at *p* < 0.05.

## 3. Results

### 3.1. Participants Characteristics

The participant characteristics are summarized in Table 1. Their maximal running speed averaged 9.52 ± 0.56 m·s^−1^, with a stride cycle frequency of 2.35 ± 0.12 Hz and a stride length of 4.36 ± 0.12 m.

### 3.2. Feasibility of Continuous sEMG Signal Acquisition in Sprinting

**Figure 4:**
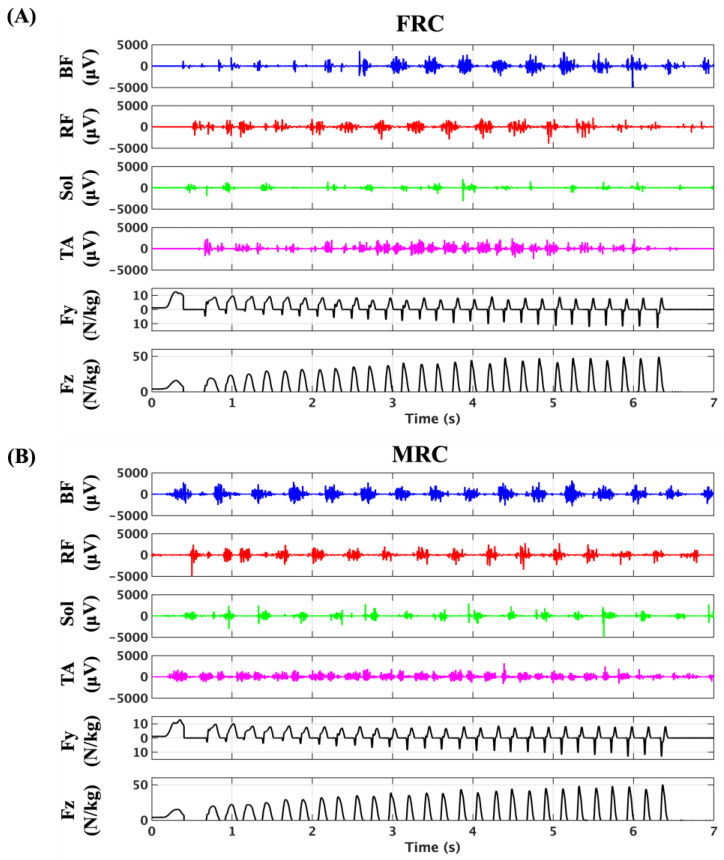
Representative recordings of sEMG (BF, RF, Sol, TA) and ground reaction force (Fy: anterior–posterior; Fz: vertical) for participant #03 during sprinting under the fixed-receiver condition (FRC, (**A**)) and mobile-receiver condition (MRC, (**B**)).

**Figure 5:**
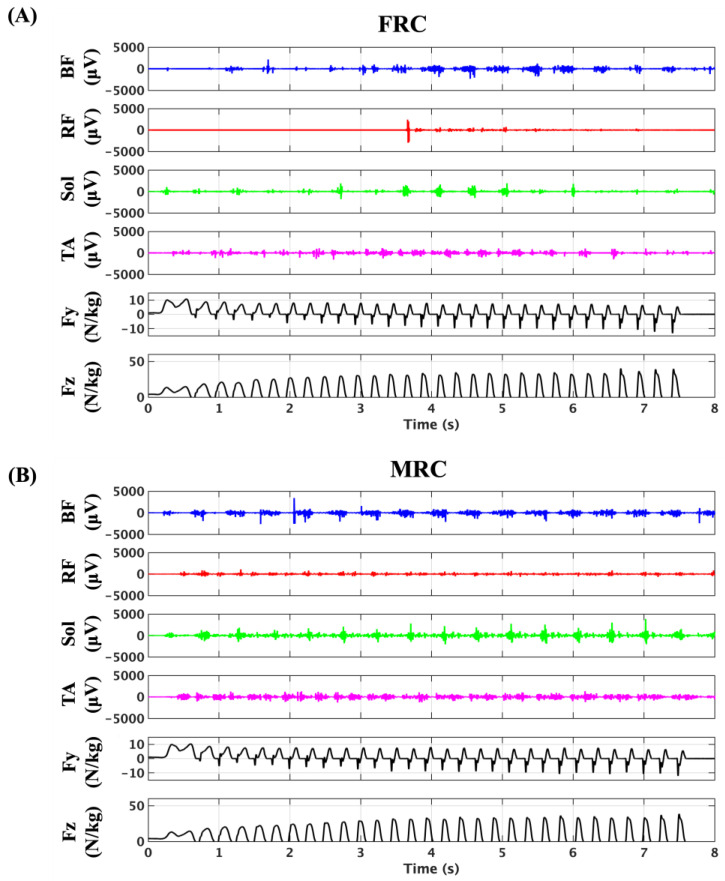
Representative recordings of sEMG (BF, RF, Sol, TA) and ground reaction force (Fy: anterior–posterior; Fz: vertical) for participant #05 during sprinting under the fixed-receiver condition (FRC, (**A**)) and mobile-receiver condition (MRC, (**B**)).

Figure 4 and Figure 5 present the sEMG and GRF data for participants #03 and #05 under the two trial conditions: fixed-receiver condition (FRC) and mobile-receiver condition (MRC). Under FRC, the sEMG signal amplitudes were markedly lower across all muscles. Specifically, the biceps femoris (BF) signals remained reduced until approximately 3 s after the start (~17 m). The rectus femoris (RF) and tibialis anterior (TA) signals were reduced until approximately 2 s (~8 m) and again from ~5 s (~35 m) onward. Soleus (Sol) signals showed consistently low amplitudes throughout the trial (Figure 4, participant #03). For participant #05, the sEMG signals of BF were reduced until approximately 4 s (~22 m) and again from ~7 s (~46 m). sEMG signals of RF were undetectable until ~3.6 s (~19 m). The sEMG signals of the Sol showed consistently low amplitudes throughout the trials. The sEMG signals of the TA were reduced until approximately 3 s (~14 m) and again from approximately 5 s (~30 m) (Figure 5). In contrast, under the MRC, all muscle signals were clearly detectable across the full sprint distance, with no major reductions observed in Figure 4 and Figure 5 (participant #03 and #05, respectively).

### 3.3. Kinetics and Kinematics Data

**Figure 6:**
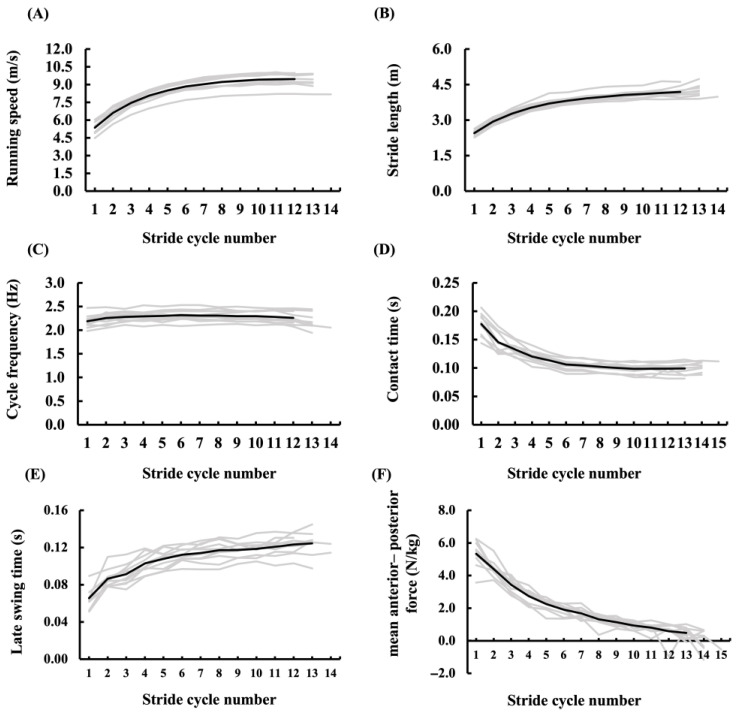
Ground reaction force (GRF) data across the stride cycles of the right leg in all participants. Panels (**A**)–(**E**) display running speed, stride length, cycle frequency, contact time, and late swing time across stride cycles; panel (**F**) shows the mean anterior–posterior force (mAP) across step numbers. Gray lines represent individual participants, and the black line represents the mean.

The cycle frequency (CF), stride length (SL), running speed (RS), and mean anteroposterior force (mAP) values for each right-leg stride cycle of all participants are shown in Figure 6. Across all participants, the mAP reached its maximum during the first step (5.39 ± 0.64 N/kg) and then decreased exponentially with increasing step number, reaching 0.41 ± 0.45 N/kg by step 13. In contrast, RS and SL increased logarithmically, with an inflection point observed at steps 4–6. The CF showed a progressive increase over the initial steps and subsequently stabilized.

### 3.4. Muscle Activities During Sprinting

**Figure 7:**
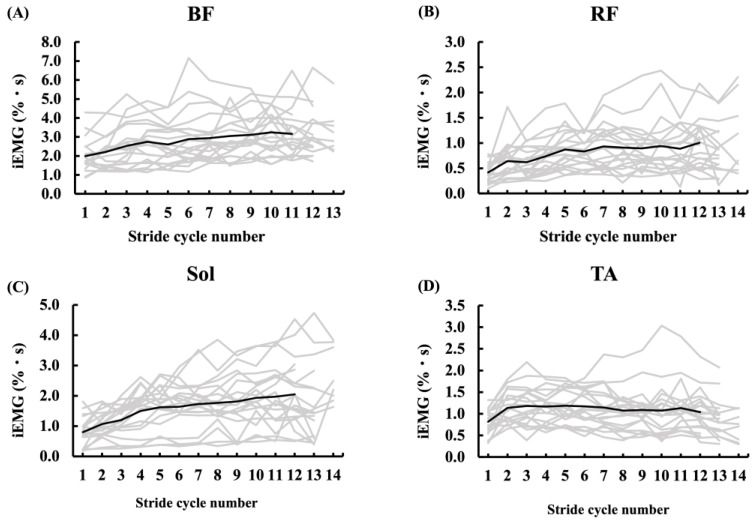
Stride-by-stride changes in integrated EMG (iEMG, expressed in %MVC·s) for the BF (**A**), RF (**B**), Sol (**C**), and TA (**D**) muscles. iEMG was obtained by time-integrating normalized EMG (%EMG@MVC) within each stride cycle. The thin gray lines represent individual participants, and the thick black lines represent the group mean.

During the 50 m sprint, the iEMG values of the BF, RF, Sol, and TA were calculated for each stride cycle under the MRC (Figure 7). Notably, the BF, RF, and Sol muscles showed a progressive increase in activity across successive stride cycles, whereas the TA muscle exhibited relatively stable activity levels from the third cycle onward. Among the four muscles analyzed, BF demonstrated the highest activation, followed by Sol, TA, and RF in descending order.

#### 3.4.1. Relationship Between Muscle Activity and Sprint Performance Variables

**Figure 8:**
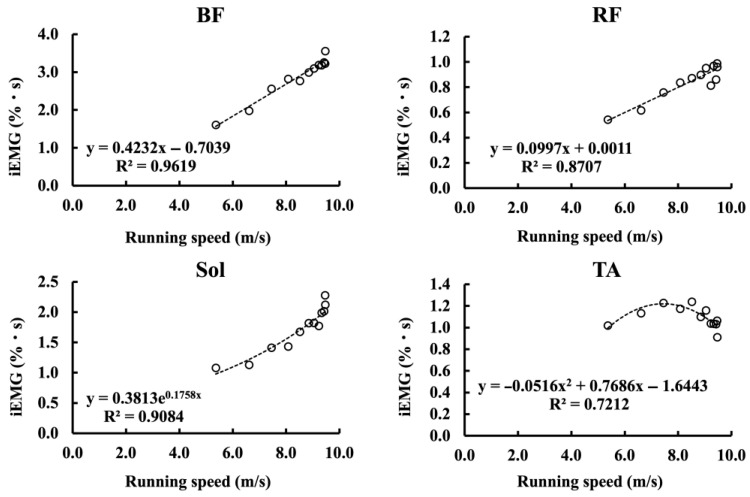
Relationships between running speed and iEMG (%MVC·s) of BF, RF, Sol, and TA, averaged across participants and plotted per stride cycle. Linear models best described BF and RF, whereas Sol showed an exponential rise above 8–9 m/s, and TA exhibited a quadratic decline beyond this threshold.

**Figure 9:**
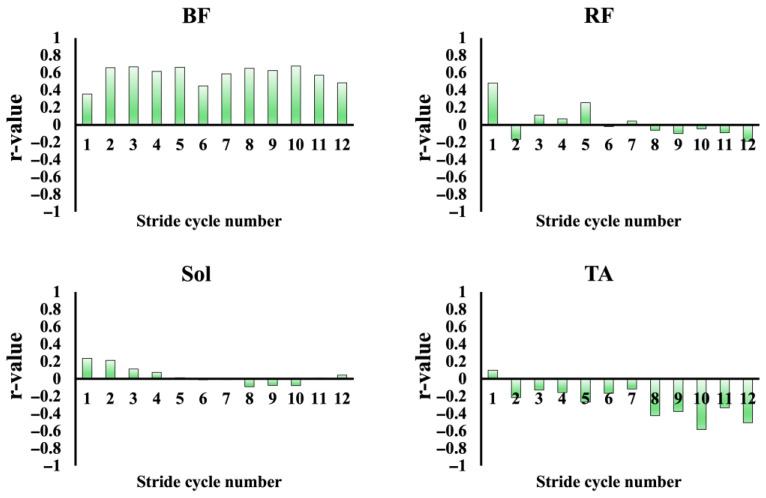
Correlation coefficients between the mAP and iEMG values during the late swing phase: BF, RF, Sol, and TA. The bars represent the correlation coefficients (r values) for each stride cycle of the right leg.

**Figure 10:**
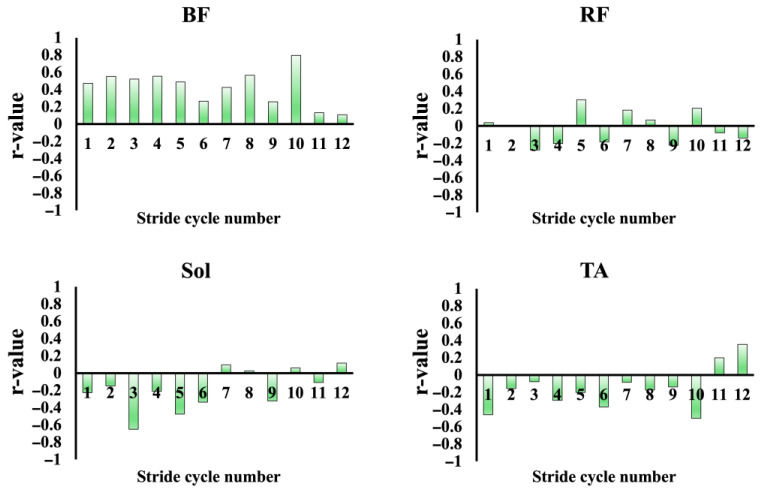
Correlation coefficients between RS and iEMG values during the late swing phase: BF, RF, Sol, and TA. The bars represent the correlation coefficients (r values) for each stride cycle of the right leg.

Figure 8 illustrates the relationships between running speed and iEMG values of each muscle, plotted for each stride cycle and averaged across all participants. For BF and RF, linear regression provided the best fit, with significant positive correlations (BF: r = 0.98, *p* < 0.05; RF: r = 0.93, *p* < 0.05). In contrast, Sol was best described by an exponential model (R^2^ = 0.91), showing a marked and sharp rise in iEMG once the running speed exceeded 8–9 m/s. The TA exhibited a convex quadratic relationship with running speed (R^2^ = 0.72), characterized by a pronounced decline in iEMG beyond 8–9 m/s. In all cases, the residual analysis confirmed the absence of systematic patterns. Furthermore, for each stride cycle, correlations were calculated between the iEMG activity of each muscle and mAP, as well as between the iEMG activity of each muscle and running speed. The resulting r-values were plotted as bar graphs (Figure 9 and Figure 10). A significant positive correlation between the iEMG activity of the BF and running speed was observed in all cycles, except for cycles 8 and 9 (*p* < 0.05). In contrast, the iEMG activity in the TA tended to show predominantly negative correlations with both running speed and mAP across most cycles, although these associations were not statistically significant. No significant correlations were observed in the other muscles.

#### 3.4.2. Muscle Activation Pattens of Agonist and Antagonist Muscles

**Figure 11:**
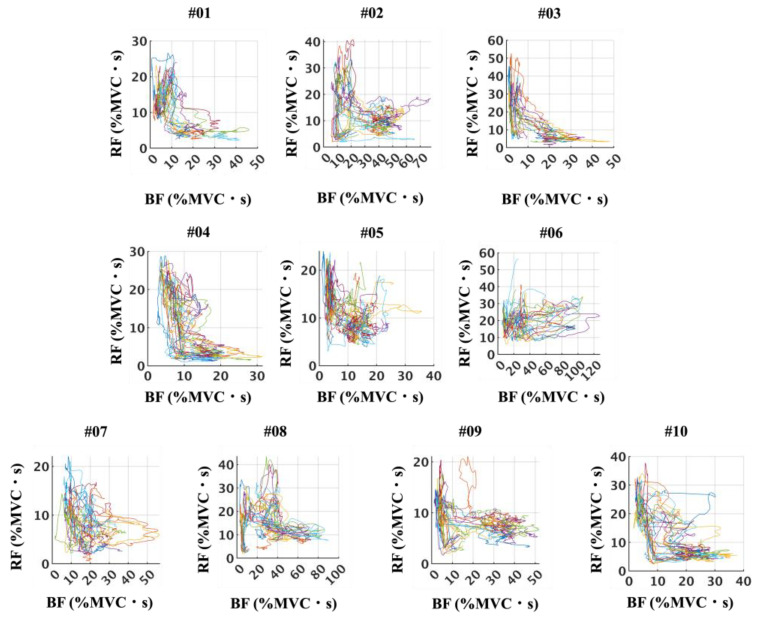
Stride-by-stride relationships between BF and RF iEMG (%MVC·s), illustrated as phase-plane plots for all participants. These plots highlight participant-specific patterns in BF–RF activation, showing considerable variability across individuals rather than a uniform trend.

**Figure 12:**
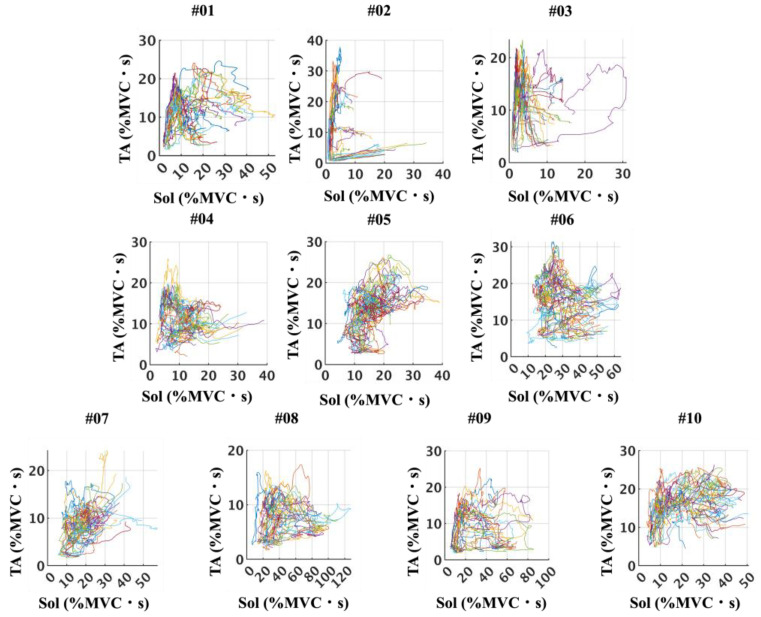
Stride-by-stride relationships between Sol and TA iEMG (%MVC·s), illustrated as phase-plane plots for all participants. These plots highlight participant-specific patterns in Sol–TA activation, showing considerable variability across individuals rather than a uniform trend.

Phase-plane plots of BF and RF iEMG (%MVC·s) (Figure 11) revealed considerable diversity in the activation patterns across participants. In some cases, the trajectories exhibited a clear reciprocal relationship, forming distinct anti-proportional shapes, whereas in others, the trajectories appeared more rounded or elliptical, suggesting less pronounced reciprocity. Similarly, phase-plane plots of Sol and TA iEMG (%MVC·s) (Figure 12) demonstrated variability among participants. While some participants showed patterns consistent with alternating activity between the Sol and TA, others displayed less distinct reciprocal features. Across all participants, no trajectories presented a strictly proportional relationship, underscoring that antagonistic coupling was variable but not characterized by direct proportionality.

**Figure 13:**
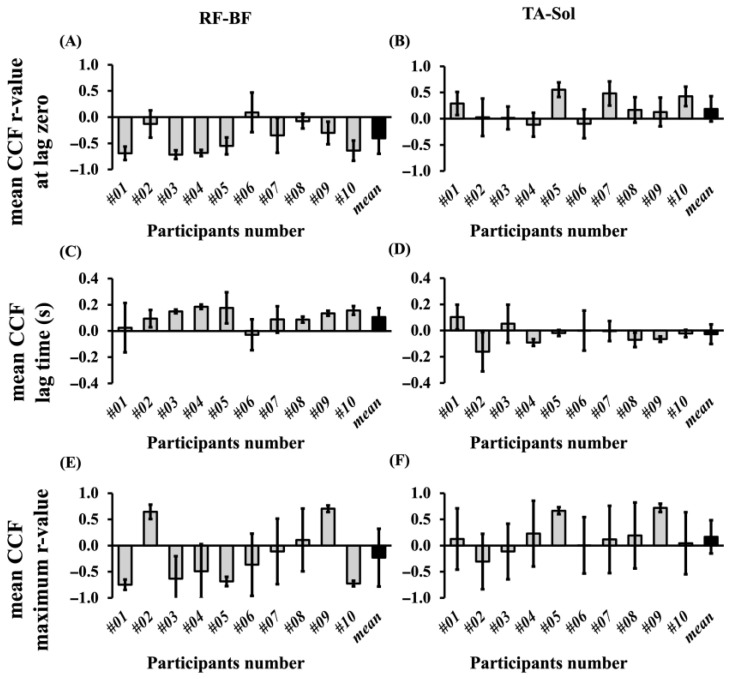
CCF values of RF–BF and TA–Sol muscle pairs over stride cycles for all the participants. CCF values are shown for the RF–BF pair in panels (**A**), (**C**), and (**E**) and for the TA–Sol pair in panels (**B**), (**D**), and (**F**). The gray lines indicate the individual participant values, and the black line represents the mean across all participants.

**Figure 14:**
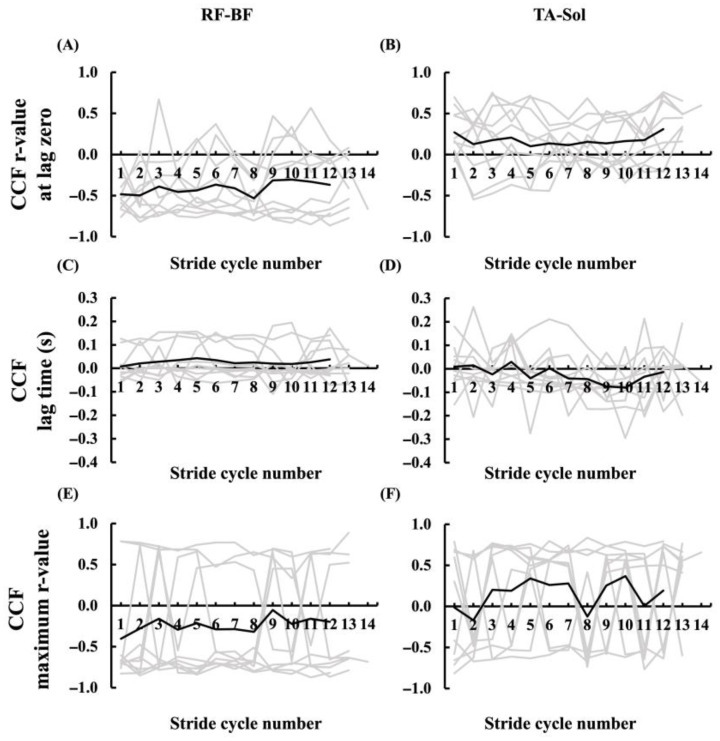
Mean CCF values of RF–BF and TA–Sol muscle pairs across all stride cycles for each participant’s right leg. Panels (**A**), (**C**), and (**E**) show the values for the RF–BF pair, and panels (**B**), (**D**), and (**F**) show those for the TA–Sol pair. Both participant-specific and average values are presented. The gray lines indicate the individual participant values, and the black line represents the mean across all participants.

The CCF between RF and BF and TA–Sol was calculated across stride cycles, and three parameters were extracted: the r-value at lag zero, lag time, and the maximum r-value. The CCF values for each stride cycle in individual participants are presented in Figure 13, and the average values across all stride cycles for each participant are shown in Figure 14, where the mean values from the two trials are displayed as bar graphs with standard deviations indicated by error bars. Across all participants, the ranges of the three CCF parameters were as follows: for the r-value at lag zero, 0.262–0.928 in RF–BF and 0.395–0.980 in TA–Sol; for lag time, −0.044 to 0.218 in RF–BF and −0.282 to 0.153 in TA–Sol; and for the maximum r-value, 0.706–0.942 in RF–BF and 0.564–0.980 in TA–Sol. The mean r-value at lag zero was 0.645 ± 0.124 in the RF–BF and 0.826 ± 0.095 in the TA–Sol, indicating lower values in the RF–BF. The mean lag time was 0.098 ± 0.008 in RF–BF and –0.019 ± 0.037 in TA–Sol, with lower values observed in the latter group. The maximum r-value was 0.842 ± 0.011 in RF–BF and 0.838 ± 0.009 in TA–Sol, showing similar results between the two pairs of muscles.

## 4. Discussion

Our main findings were as follows: (1) Although reliable transmission of sEMG signals was limited when the recording distance exceeded the effective range of the device, continuous and high-quality sEMG recordings over the entire 50-m sprint were feasible under the mobile-receiver condition (MRC). (2) BF activity during the late swing phase was significantly positively associated with sprint velocity, supporting the functional relevance of the pawing action to sprint performance. (3) Coordination analyses revealed distinct patterns of agonist–antagonist interactions: reciprocal activation was predominant in the BF/RF, whereas coactivation was predominant in the Sol/TA. These findings highlight both the performance-related benefits and the stabilization strategies employed during sprinting.

### 4.1. Wireless Transmission–Related Attenuation of sEMG Signals

In the fixed-receiver condition (FRC), where the receiver was positioned at the 25-m midpoint of the track, all four sEMG signals exhibited marked attenuation once the runner moved beyond the effective transmission range (approximately 20 m). This resulted in intermittent or absent signals until the runner came closer to the receiver. In contrast, in the mobile-receiver condition (MRC), where the receiver was dynamically maintained within an appropriate distance from the runner, continuous and reliable sEMG recordings were obtained throughout the full 50-m sprint. These results confirm that the transmission range of the device is a primary limiting factor for wireless EMG acquisition, rather than environmental interference [1]. Importantly, they also highlighted the practical importance of adapting receiver placement strategies in applied sprint experiments to ensure high-quality signal capture under field conditions.

### 4.2. Relationship Between Muscle Activity and Sprint Performance Variables

A significant positive correlation was observed between BF iEMG in the late swing (LS) phase and the sprint velocity. The LS phase corresponds to the backward swing of the leg in the air, often referred to as “pawing”. Previous studies have reported that the hamstrings experience substantial loading in this phase, sometimes exceeding that of the stance phase [8,22,23,24,25], thereby elevating the risk of injury [26]. The absence of a significant correlation between BF activity in the LS and mAP is consistent with previous studies that found limited evidence that pawing directly generates a propulsive force at ground contact [3,27]. However, our results indicate that pawing is positively associated with sprint velocity, supporting the view that it contributes to performance rather than being detrimental. This implies that pawing may enhance swing-phase mechanics, such as faster backward swing velocity and rapid limb repositioning, rather than directly generating propulsive force at ground contact. Reportedly, a positive correlation between sprint velocity and hip extension velocity in the LS in elite athletes [28], suggesting that higher sprint speeds may be associated with a rapid backward swing driven by BF activation. Nevertheless, the physiological origin of BF activity observed during the late swing (LS) phase remains unclear. While one possibility is that it reflects stretch reflex contributions, our study did not directly assess reflex responses, making this interpretation speculative. The central descending drive and spinal rhythmogenic mechanisms represent alternative explanations. Evidence from animal models provides further insight: in cats, paw-shake responses selectively recruit fast-type motor units [29], with central pattern generators (CPGs) providing the main rhythmic drive, while sensory feedback acts as a modulator rather than the primary source [30]. Neuromechanical studies have further shown that paw-shake accelerations emerge from the interaction of spinal CPG output, segmental inertia, and viscoelastic muscle properties [31], with intersegmental energy transfer amplifying whip-like oscillations [32]. Human studies parallel these findings, showing the selective recruitment of fast-type motor units at high angular velocities [33,34]. These results suggest that LS-phase BF activity during sprinting likely reflects an integration of the central rhythmic drive, velocity-dependent motor unit recruitment, and passive biomechanical amplification. This multifactorial interpretation underscores that LS-phase EMG activity should not be viewed solely as a reflex. Importantly, while greater BF activation may benefit sprint performance by supporting rapid pawing, it also coincides with a phase of high eccentric strain, indicating a trade-off between performance gains and an elevated hamstring injury risk. Future studies should clarify how athletes can optimize this balance.

In summary, BF activity in the LS phase appears to emerge from an interplay of central drive, velocity-dependent recruitment, and passive biomechanical amplification rather than being attributable to a single reflexive mechanism. This multifactorial view underscores the need to move beyond single-muscle interpretations and examine how groups of muscles interact to achieve both performance and stability.

### 4.3. Agonist–Antagonist Coordination Patterns

Our analyses of the BF/RF and Sol/TA muscle pairs highlighted distinct neuromuscular strategies during sprinting. The BF/RF pair demonstrates considerable inter-individual variability, with some athletes showing reciprocal activation consistent with efficient alternation of hip and knee torques [8,22,23,24,25], and others showing more coactivation, potentially reflecting a stabilizing strategy under high angular velocities [4,5,16]. This variability underscores the individualized nature of femoral muscle coordination, suggesting that athletes may adopt different neural strategies to balance propulsion efficiency and joint protection. In contrast, the Sol/TA pair showed consistently higher simultaneous activation across participants, indicating a common strategy of ankle stiffening during the late swing phase to prepare for a stable ground contact [25]. This uniformity suggests that ankle stabilization is a fundamental requirement for sprinting and is less subject to individual variation than femoral muscle coordination. From a neurophysiological perspective, these findings illustrate the dual roles of agonist–antagonist interactions: (1) individualized strategies at the thigh level, where reciprocal inhibition versus coactivation reflects a trade-off between efficient torque generation and stability [4,5,16], and (2) a shared stabilizing role at the ankle, where coactivation ensures consistent readiness for impact [25]. From a performance perspective, monitoring BF/RF coordination patterns may help identify whether an athlete relies more on efficient alternation or stabilizing coactivation during performance. Excessive coactivation may hinder propulsion, whereas highly reciprocal patterns may reduce stability under load. From an injury prevention perspective, sustained femoral muscle coactivation may elevate hamstring loading in LS, thereby increasing the risk of injury [4,5,16,22].

Future studies should build on these findings by linking coordination profiles with sprint performance outcomes and injury history. Longitudinal monitoring may reveal whether coordination strategies are stable traits or adaptable through training. Such studies could clarify whether individual variability in BF/RF coordination represents adaptive optimization or compensatory patterns that predispose athletes to injury.

### 4.4. Limitations

This study had several limitations. The small sample size and restriction to the right-leg superficial muscles limit generalizability and may overlook asymmetries or deeper muscle contributions. In addition, a post hoc sensitivity analysis (α = 0.05, power = 0.80, two-tailed; G*Power) indicated that with the present sample size (*n* = 10), only strong correlations (|r| ≥ 0.76) could be detected, meaning that moderate-to-small associations may not have been captured. Furthermore, this study did not systematically investigate wireless transmission parameters such as packet loss, maximum transmission distance, or optimal sampling frequency. The present study was restricted to demonstrating the feasibility of continuous wireless EMG recordings during sprinting and analyzing neuromuscular coordination patterns. The evaluation of wireless transmission was confined to two receiver configurations, and the analytical methods were correlational, indicating that causal links between neural control and performance remain to be established. Furthermore, the exploratory subgroup analysis between 100-m sprinters (*n* = 4) and 400-m hurdlers (*n* = 3) should be interpreted with caution, as the very small subgroup sizes preclude generalization and the findings should be regarded as preliminary. Future studies should combine wireless EMG with advanced biomechanical and neurophysiological tools, such as motion capture, high-density EMG, and brain stimulation methods, to provide a more comprehensive understanding of sprint-specific neuromuscular control.

## 5. Conclusions

This study confirmed that continuous and reliable sEMG recordings during a 50-m sprint are feasible when using a mobile-receiver configuration, whereas a fixed-receiver setup resulted in substantial signal loss. Physiological analyses revealed that BF activity during the late swing phase was positively associated with sprint velocity, underscoring its functional role in rapid backward leg motion and highlighting potential links to injury risk. Coordination analyses further showed that BF/RF activation patterns varied across individuals, reflecting diverse neuromuscular strategies, whereas Sol/TA pairs consistently exhibited coactivation, suggesting a stabilizing function of the ankle joint. These findings indicate that EMG activity during sprinting reflects both individualized and common coordination strategies. Wireless EMG systems thus provide a practical tool for monitoring agonist–antagonist interactions in applied environments, with applications in both performance optimization and injury prevention in sprinting athletes.

## Figures and Tables

**Table 1:** Individual participant characteristics (anthropometric and maximal sprint performance variables) with corresponding group averages and standard deviations.

Participant Number	Height (m)	Body Mass (kg)	Age (yr)	Maximal Running Speed (m·s^−1^)	Maximal Cycle Frequency (Hz)	Maximal Cycle Stride Length (m)	Maximal Mean Anteroposterior (N/kg)	Sprint Event	Personal Record (in Seconds)
#01	1.732	65.3	27	9.18	2.36	4.22	5.18	110 mH	15.49
#02	1.663	64.4	26	10.05	2.46	4.37	5.99	100 m	10.53
#03	1.724	74.0	25	10.02	2.42	4.30	5.27	200 m	21.42
#04	1.708	64.7	21	9.30	2.26	4.74	6.20	400 mH	58.1
#05	1.628	59.3	22	8.23	2.13	4.06	5.28	400 mH	64.28
#06	1.714	66.1	19	9.07	2.38	4.25	4.77	110 mH	15.74
#07	1.629	68.0	21	9.91	2.53	4.18	5.52	100 m	10.83
#08	1.678	68.9	20	9.92	2.46	4.32	5.38	100 m	11.46
#09	1.828	71.0	19	10.01	2.24	4.74	6.50	100 m	11.16
#10	1.77	66.6	18	9.48	2.29	4.45	5.85	400 mH	53.32
mean	1.707	66.83	21.8	9.52	2.35	4.36	5.59		
SD	0.059	3.80	2.99	0.56	0.12	0.21	0.50		

## Data Availability

Data are contained within the article; please contact the authors if you need anything else.
